# Comparison of Predictive Models for Keloid Recurrence Based on Machine Learning

**DOI:** 10.1111/jocd.70008

**Published:** 2025-02-07

**Authors:** Yan Hao, Mengjie Shan, Hao Liu, Yijun Xia, Xinwen Kuang, Kexin Song, Youbin Wang

**Affiliations:** ^1^ Department of Plastic and Cosmetic Surgery Peking Union Medical College Hospital Beijing China; ^2^ Peking Union Medical College, Chinese Academy of Medical Sciences Peking Union Medical College Hospital Beijing China

**Keywords:** keloid, machine learning, prediction model, recurrence, risk factors

## Abstract

**Objectives:**

To establish, evaluate and compare three recurrence prediction models for keloid patients using machine learning methods.

**Methods:**

We enrolled 301 keloid patients who underwent surgery and postoperative radiotherapy, dividing them into a training set (70%) and a validation set (30%). Three recurrence prediction models were established in the training set: the logistic regression model, the decision tree model, and the random forest model. We then evaluated and compared the performance of these models in the validation set, using metrics such as accuracy, sensitivity, specificity, recall, precision, kappa coefficient, and the area under the ROC curve (AUC).

**Results:**

We developed three machine learning‐based prediction models for keloid recurrence. KAAS, mean arterial pressure levels, postoperative complications, and the proportion of inflammatory cells played crucial roles in these models. The decision tree model outperformed both the random forest and logistic regression models in terms of accuracy, and it also exhibited the highest overall precision. Regarding AUC, logistic regression performed the best, followed by random forest and decision trees.

**Conclusions:**

This study established three prediction models for keloid recurrence using machine learning techniques, highlighting the significance of KAAS, blood pressure levels, postoperative complications, and inflammatory cell proportions. When compared from various dimensions, the logistic regression model demonstrated the most favorable prognostic performance in terms of AUC.

## 1 Introduction

Keloid, a common benign skin condition characterized by excessive fibroblast proliferation and collagen deposition, frequently develops as a result of trauma, surgery, or folliculitis [1]. The incidence rate during the wound healing process ranges from 5% to 15% [2]. The prevailing treatment strategy involves a combination of surgery followed by postoperative radiation therapy. However, there is still a certain degree of recurrence rate, which significantly impacts the quality of life for patients [3]. Unfortunately, predictive models for keloid recurrence are scarce in current research. In this study, we leverage clinical data to establish and compare three predictive models for keloid recurrence using machine learning (ML) techniques. Our objective is to gain deeper insights into the factors influencing keloid recurrence.

ML is a subset of artificial intelligence that has gained significant popularity in recent years. Broadly speaking, ML refers to computationally intensive methods that employ data‐driven approaches to develop models. In comparison to traditional modeling techniques, ML requires fewer modeling decisions from the modelers [4, 5, 6]. ML models can be categorized into several types, including supervised learning models. Supervised learning models require annotated training data to learn the relationships between data points and make predictions on unknown data. For example, supervised learning models can be used for classification problems and regression problems. Unsupervised learning models, on the other hand, do not require annotated training data. They can perform operations like clustering and dimensionality reduction to discover inherent relationships among data points, addressing problems such as clustering and dimensionality reduction [7]. ML algorithms have been widely applied in the prognosis and prediction of serious diseases such as sepsis, gastrointestinal bleeding, pneumonia, acute poisoning, and chronic obstructive pulmonary disease (COPD) [8, 9, 10, 11, 12, 13, 14].

Predicting disease recurrence is a binary classification problem, and several models are commonly used for this purpose. Logistic Regression: Logistic regression is a simple and effective classification model used for modeling binary classification problems. It can predict whether a disease will recur based on various patient factors such as age, gender, medical history, etc. The advantages of logistic regression models include high computational efficiency, interpretability, the ability to identify risk factors, and the possibility of visualization through probability plots [15]. Decision tree is a classification model based on a tree‐like structure. It can handle nonlinear data and classify data through a series of binary decisions, dividing the data into multiple subsets, with each subset representing a decision tree node. Decision tree models can be used to explain the decision‐making process of the model, are easy to understand, and can be presented and interpreted using visualization tools. This makes it more intuitive to understand the structure and attributes of the decision tree. Additionally, decision tree models can be adjusted and optimized as needed [16]. Random forest is an ensemble model based on decision trees. It creates multiple decision trees by randomly selecting features and samples, and then makes predictions by voting or averaging the results from these trees. It can handle high‐dimensional data and is particularly robust in dealing with noisy data [17].

The choice of which model to employ is contingent upon factors such as data characteristics, feature dimensionality, dataset size and complexity, as well as accuracy requirements. Generally, it is recommended to conduct a comparative analysis using multiple diverse models and select the one that best suits the research objectives. We will determine the optimal model by conducting a comparative analysis of three distinct predictive models for keloid recurrence subsequent to surgical intervention and postoperative radiation therapy. This assessment will encompass various performance metrics, including accuracy, sensitivity, specificity, recall, precision, and area under the receiver operating characteristic (ROC) curve (AUC), along with their associated 95% confidence intervals (CI).

## 2 Methods

### 2.1 Patient Characteristics

During the period from January 2015 to January 2019, clinical data were gathered from 399 patients who had been admitted to the Department of Plastic and Cosmetic Surgery at Peking Union Medical College Hospital for the treatment of keloids. Following thorough follow‐up through telephone interviews and outpatient visits, patients with incomplete or low‐quality follow‐up data were excluded, resulting in the inclusion of 301 patients in the study. Postoperative radiation therapy was administered using electron beam radiation therapy, delivering a cumulative dose of 18 Gy divided into two fractions, each delivering 9 Gy. The first radiation fraction was administered 24 h after surgery, with the second fraction given after a one‐week interval. The study collected data on 17 predictor variables, which included gender, age, surgical procedure, whether it was the initial treatment, history of infections, whether the etiology was iatrogenic, surgical site, postoperative complications, mean arterial pressure (MAP), body mass index (BMI), keloid activity assessment scale (KAAS), preoperative white blood cell count, lymphocyte percentage, neutrophil percentage, monocyte percentage, eosinophil percentage, and hemoglobin levels. The outcome variable under consideration was the occurrence of recurrence within a 2‐year timeframe. General clinical information is summarized in Table 1.

**TABLE 1:** Characteristics of the patients and keloids.

Categorical variable	Groups	*n*	Recurrence(*n*)	Non‐recurrence(*n*)	Rate of recurrence
Gender	Male	119	41	78	0.344
	Female	182	57	125	0.313
Surgical procedure	Local flap	143	42	101	0.294
	Direct excision	74	25	49	0.338
	Expander	29	12	17	0.414
	Perforator flap	18	9	9	0.5
	Core excision	7	2	5	0.286
	Keloid flap	23	4	19	0.174
	Skin grafting	5	2	3	0.4
	Free flap	2	2	0	1
Initial treatment	Yes	135	41	94	0.304
	No	166	57	109	0.343
History of infections	Yes	40	14	26	0.35
	No	261	84	177	0.322
Etiology	Iatrogenic	59	9	50	0.153
	Non‐iatrogenic	242	89	153	0.368
Surgical site	Abdomen	19	2	17	0.105
	Back	22	6	16	0.273
	Chest	145	52	93	0.359
	Ear	23	4	19	0.174
	Head	23	6	17	0.261
	Limbs	22	10	12	0.455
	Perineum	21	9	12	0.429
	Shouder	26	9	17	0.346
Postoperative complications	Yes	20	15	5	0.75
	No	281	83	198	0.295
Numerical variable	Groups			median(IQR)	
Mean arterial pressure (MAP)	Recurrence			92.7 (84.9, 98.7)	
	Non‐recurrence			91.7 (85.3, 98.3)	
BMI	Recurrence			23.3 (21.5, 27.7)	
	Non‐recurrence			23.8 (21.9, 26.4)	
KAAS	Recurrence			6 (5, 7)	
	Non‐recurrence			4 (3, 5)	
White blood cell count	Recurrence			6.2 (5.5, 7.4)	
	Non‐recurrence			5.9 (5.1, 7.3)	
Neutrophil percentage	Recurrence			57.7 (7.2)	
	Non‐recurrence			55.4 (7.8)	
Lymphocyte percentage	Recurrence			33.2 (6.9)	
	Non‐recurrence			34.3 (7.6)	
Monocyte percentage	Recurrence			5.4 (4.6, 6.7)	
	Non‐recurrence			5.4 (4.6, 6.3)	
Eosinophil percentage	Recurrence			2.1 (1.6, 3.3)	
	Non‐recurrence			1.9 (1.4, 3.1)	
Hemoglobin	Recurrence			138.5 (128.5151)	
	Non‐recurrence			136 (128149.5)	
Age	Recurrence			28 (24, 38.8)	
	Non‐recurrence			31 (25, 46)	

### 2.2 Statistical Analysis

All statistical analysis, model building, and visualization were conducted using the R software package (version 4.1.3, Vienna, Austria). The ‘caret’ package's ‘createDataPartition’ function was employed to randomly split the dataset into a training set (70%) and a testing set (30%).

#### 2.2.1 Logistic Regression Model Building

The ‘tableStack’ function from the ‘epiDisplay’ package was used to conduct univariate analysis on all predictor variables in the training set. Variables with *p* < 0.05 were selected as feature variables. The ‘dplyr’ package and ‘GGally’ package were employed to visualize the correlations among the selected feature variables. The ‘rms’ package was used to perform multivariate regression analysis on the feature variables in the training set, and variables with *p* < 0.05 were included in the logistic regression model.

#### 2.2.2 Decision Tree Model Building

The ‘rpart’ package was utilized to build a decision tree model in the training set. To reduce overfitting, the ‘prune’ function was applied to the initial model. The ‘rpart.plot’ function was used for visualizing the decision tree model.

#### 2.2.3 Random Forest Model Building

The ‘randomForest’ package was employed to build a random forest model in the training set. The ‘varImpPlot’ function was used to create a plot illustrating the ranking of variable importance.

#### 2.2.4 Comparison and Evaluation of the Three Models

The models were evaluated and compared in the test dataset. The ‘caret’ package was used to assess model performance through confusion matrices, calculating accuracy, sensitivity, specificity, recall, precision, and kappa coefficient for the three models. Additionally, the ‘pROC’ package was employed to compute the AUC and create ROC curves for the three models. The best‐performing model was evaluated using the bootstrapping method.

### 2.3 Calculation of the Confusion Matrix

TP (true positive): The number of correctly predicted positive instances.

FP (false positive): The number of negative instances incorrectly predicted as positive.

FN (false negative): The number of positive instances incorrectly predicted as negative.

TN (true negative): The number of correctly predicted negative instances.

Accuracy: This metric represents the probability of correctly predicting all samples and is calculated as (TP + TN)/(TP + FP + TN + FN). However, accuracy has limitations in cases of severe imbalance between positive and negative samples, where it may not be meaningful.

Sensitivity: It is calculated as TP/(TP + FN). It measures the proportion of actual positive samples that are correctly predicted.

Specificity: It is calculated as TN/(FP + TN). It measures the proportion of actual negative samples that are correctly predicted.

Recall: It is calculated as TP/TN + TP. The proportion of all positive samples that are correctly predicted among those predicted as positive is commonly referred to as recall.

Precision: It is calculated as TP/(TP + FP). It measures the proportion of correctly predicted positive samples among those identified as positive.

Kappa coefficient: The kappa coefficient assesses consistency and agreement between predicted results and actual outcomes. It is calculated on the basis of the confusion matrix and ranges from −1 to 1, with values greater than 0.4 indicating strong consistency, between 0.2 and 0.4 indicating moderate consistency, and less than 0.2 indicating poor consistency.

AUC: The AUC is defined as the area enclosed by the ROC curve and the coordinate axes. A higher AUC value, closer to 1.0, indicates higher reliability of the detection method, whereas a value of 0.5 signifies the lowest level of accuracy with no practical value.

## 3 Results

### 3.1 General Clinical Information

A total of 301 patients were analyzed. The characteristics of the patients and keloids are summarized in Table 1. The recurrence rate within 2 years after treatment in the dataset was 32.56%.

### 3.2 Establishment of a Logistic Regression Model

The data was divided into a 70% training set (*n* = 212) and a 30% testing set (*n* = 89).

Following univariate analysis on the training set, six feature variables with *p* < 0.05 were selected: age, KAAS, postoperative complications, etiology (iatrogenic or not), neutrophil percentage, and lymphocyte percentage. These variables were subsequently subjected to multivariate regression analysis (refer to Table 2).

**TABLE 2:** Univariate and multivariate analysis.

	Univariate analysis (*p*‐value)	Multivariate analysis (*p*‐value)
Gender	0.616	
Age	0.018*	0.202
Surgical procedure	0.081	
Surgical site	0.317	
History of infections	0.052	
Initial treatment	0.218	
Etiology	0.001**	0.303
Postoperative complications	< 0.001***	0.0016**
BMI	0.705	
MAP	0.294	
KAAS	< 0.001***	< 0.001***
White blood cell count	0.114	
Neutrophil percentage	0.043*	0.665
Lymphocyte percentage	0.022*	0.330
Monocyte percentage	0.75	
Eosinophil percentage	0.99	
Hemoglobin	0.761	

*0.01 ≤ *p* < 0.05; **0.001 ≤ *p* < 0.01; ***p < 0.001.

In the multivariate regression analysis, two variables, KAAS and the occurrence of postoperative complications, exhibited *p* < 0.05. Postoperative complications (Yes vs. No, OR = 11.81) and KAAS (OR = 2.03) indicated that the presence of postoperative complications and higher KAAS scores are associated with an elevated risk of recurrence.

### 3.3 Establishment of Decision Tree and Random Forest Models

**FIGURE 1:**
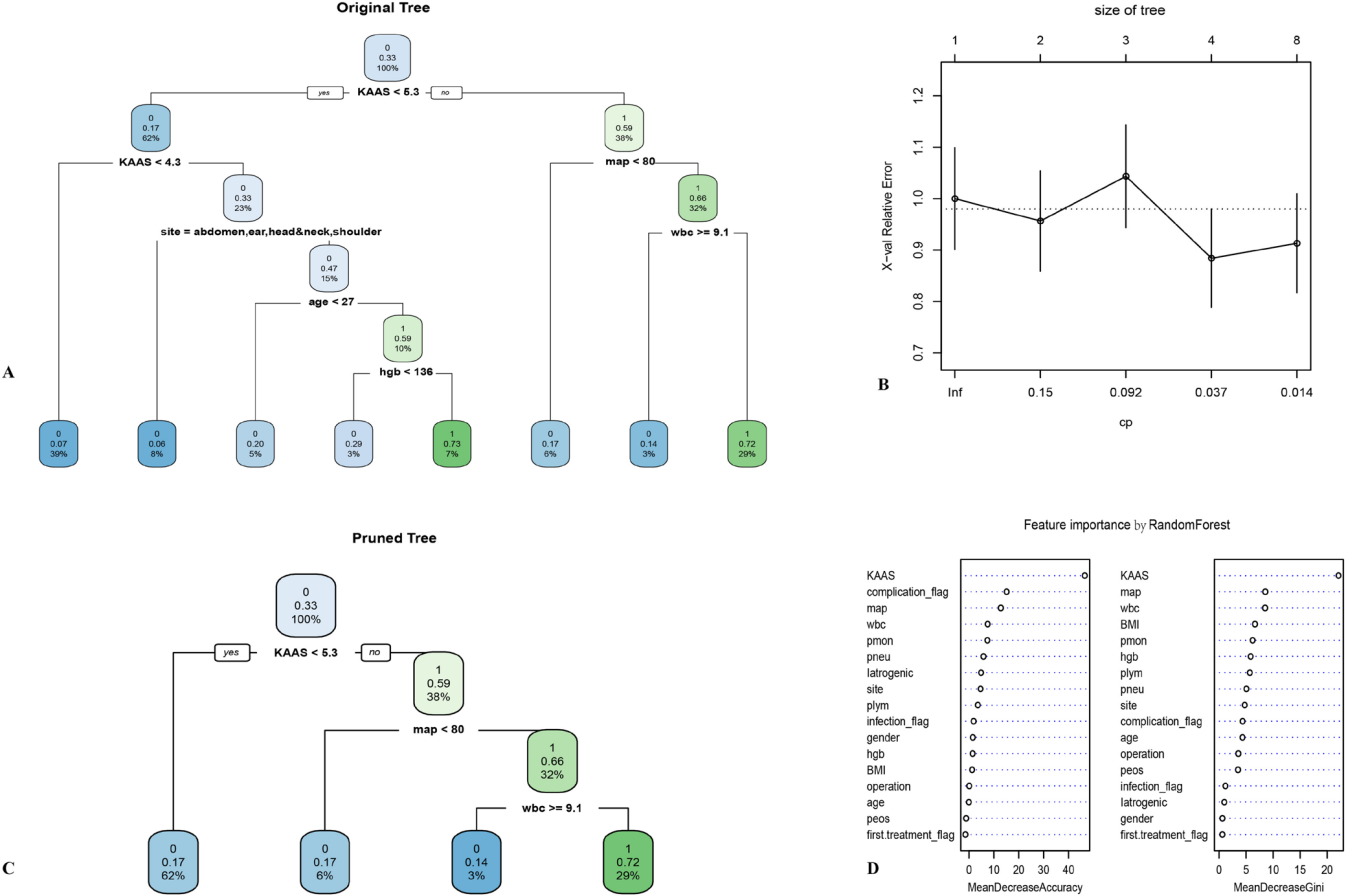
(A) Original decision tree model. (B) Identifying the minimum cross‐validation error for the classification tree model, locating the record with the minimum cross‐validation error, which is at 4. (C) Pruned decision tree model. (D) The importance of feature variables in the random forest model. complication_flag, Postoperative complication flag; first treatment_flag, first‐time treatment flag; hgb, hemoglobin; Iatrogenic, iatrogenic etiology; infection_flag, History of infection flag; KAAS, keloid activity assessment scale; map, mean arterial pressure; peos, eosinophil percentage; plym, lymphocyte percentage; pmon, Monocyte percentage; pneu, neutrophil percentage; wbc, white blood cell count.

In a decision tree model, the root node represents the entire dataset, whereas branching nodes below the root node represent specific attributes or features. Features are ranked by importance, with the most crucial features appearing higher in the ranking. Notably, KAAS emerged as the most critical feature in the decision tree prediction model. The model evaluates feature values of a sample by navigating down the corresponding branch, ultimately reaching a leaf node that signifies the model's classification result. The classification outcomes are represented as 0 (non‐recurrence) and 1 (recurrence), with percentages indicating sample proportions. The middle numerical values denote the Gini index, a performance metric for classification tree models. Smaller values indicate higher purity(Figure 1A–C). Figure 1A: Original decision tree model; Figure 1B: Identifying the minimum cross‐validation error for the classification tree model, locating the record with the minimum cross‐validation error, which is at 4. Obtaining the cost complexity parameter value for the record with the minimum cross‐validation error and setting the parameter ‘cp’ to the same value for pruning purposes; Figure 1C: Pruned decision tree model. The importance of feature variables in the random forest model is presented in Table 3 and Figure 1D.

**TABLE 3:** Importance of features in the random forest model.

Random forest importance	0	1	MeanDecrease accuracy	MeanDecrease gini
KAAS	33.872	38.567	46.519	22.111
Postoperative complications	12.984	11.419	15.122	4.348
MAP	13.442	4.433	12.810	8.548
White blood cell count	6.273	4.104	7.526	8.511
Monocyte percentage	7.116	3.116	7.422	6.200
Neutrophil percentage	6.723	0.686	5.853	5.056
Etiology	−0.659	7.730	4.872	0.955
Surgical site	5.318	1.221	4.659	4.730
Lymphocyte percentage	4.600	−0.474	3.571	5.681
History of infections	1.071	1.589	1.892	1.160
Gender	3.268	−1.979	1.548	0.607
Hemoglobin	6.135	−5.239	1.483	5.835
BMI	4.810	−4.229	1.270	6.644
Surgical procedure	2.582	−2.696	0.119	3.560
Age	1.009	−1.184	−0.036	4.326
Eosinophil percentage	1.365	−3.629	−1.103	3.502
Initial treatment	−0.903	−1.413	−1.388	0.597

### 3.4 Evaluation and Comparison of the Three Models

**FIGURE 2:**
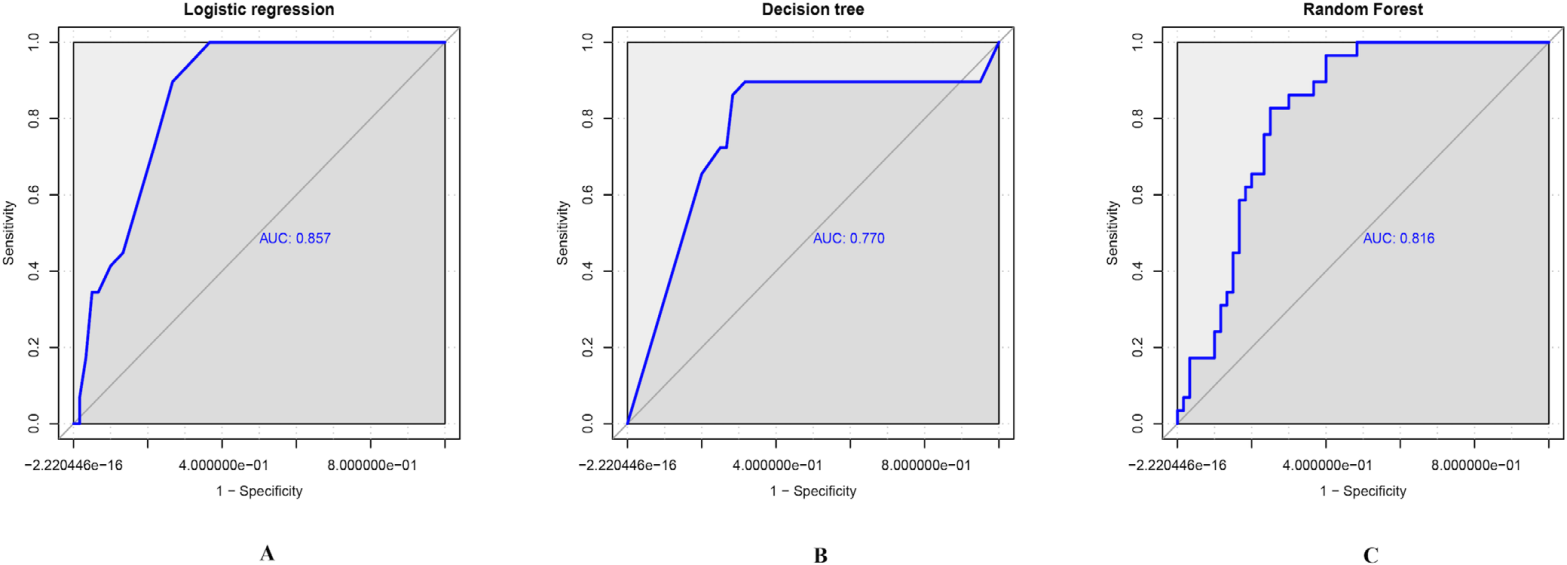
(A) ROC plot in the logistic regression model, AUC = 0.857. (B) ROC plot in the decision tree model, AUC = 0.770. (C) ROC plot in the random forest model, AUC = 0.816. AUC, area under the ROC curve; ROC, receiver operating characteristic.

Table 4 displays the values of accuracy, sensitivity, specificity, recall, precision, kappa coefficient, and AUC, along with their corresponding 95% CI for the three models. Figure 2 presents the ROC curves.

**TABLE 4:** Evaluation and comparison of the three models.

	Logistic regression	Decision tree	Random forest
Accuracy	0.730	**0.753**	0.7416
Sensitivity	0.448	**0.655**	0.5172
Specificity	**0.867**	0.8	0.8
Recall	**0.619**	0.613	0.6
Precision	0.448	**0.655**	0.621
Kappa coefficient	0.3391	**0.4472**	0.417
AUC(95% CI)	**0.857 (0.782–0.931)**	0.770 (0.657–0.883)	0.816 (0.73–0.902)

*Note:* The best values for each metric across the three models are highlighted in bold in Table 4 to facilitate comparison.

Accuracy: Among the three models, the decision tree model achieved the highest accuracy, followed by the random forest and logistic regression models.

Sensitivity: The decision tree model exhibited the highest sensitivity, indicating its ability to correctly identify positive cases, followed by the random forest and logistic regression models.

Specificity: The random forest model displayed the highest specificity, signifying its capacity to correctly identify negative cases. The logistic regression and decision tree models had similar specificity.

Recall: Logistic regression had the highest recall, indicating its effectiveness in identifying positive cases, followed by the decision tree and random forest models.

Precision: The decision tree model had the highest precision, signifying its ability to accurately predict positive cases, followed by the random forest and logistic regression models.

Kappa coefficient: The decision tree model had the highest kappa coefficient, reflecting strong consistency in its predictions. The random forest model followed, with the logistic regression model having the lowest kappa coefficient.

**FIGURE 3:**
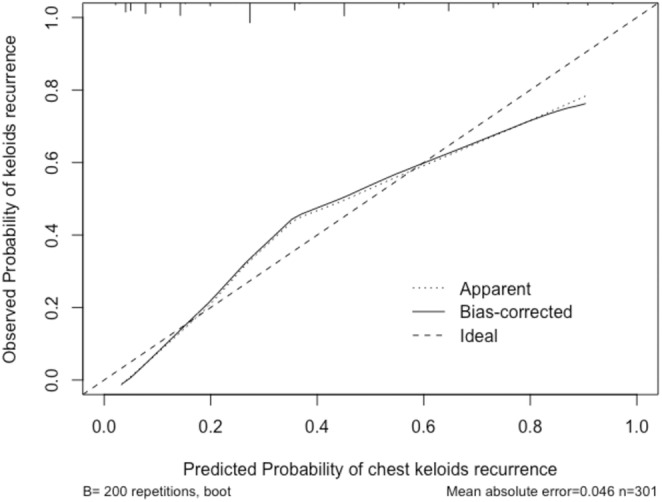
Performance evaluation of the logistic regression model using the bootstrapping method.

AUC: Logistic regression had the highest AUC (0.857), indicating the strongest overall model performance. The random forest model came next (0.816), whereas the decision tree model had the lowest AUC (0.770). We evaluated the logistic regression model using the bootstrapping method, as shown in Figure 3.

These findings provide a comprehensive comparison of the three models in terms of their predictive performance on the test dataset.

## 4 Discussion

We established three predictive models for keloid recurrence after surgical intervention and postoperative radiation therapy using ML methods. Each of these models exhibited unique strengths and varying performance metrics, shedding light on their respective roles in predicting keloid recurrence. The decision tree model demonstrated the highest accuracy and sensitivity among the three models. Its superior sensitivity implies an ability to effectively identify patients at risk of keloid recurrence. However, it's essential to note that decision trees are susceptible to overfitting due to their tendency to incorporate a multitude of variables. To reduce overfitting, pruning was performed. After pruning, the model highlighted KAAS, MAP, and white blood cell count as the most important feature variables. Our random forest model performed commendably, ranking second in both accuracy and sensitivity. Known for its robustness, it excelled in handling high‐dimensional and noisy data. Key features identified by the model included KAAS, MAP, and preoperative inflammatory status, all of which proved influential in predicting recurrence risk. Although the logistic regression model did not top the charts in accuracy or sensitivity, it distinguished itself with the highest AUC. AUC serves as a critical metric for assessing overall model performance, particularly in scenarios where balancing sensitivity and specificity is paramount. In the logistic regression analysis, univariate analysis revealed that neutrophil percentage and lymphocyte percentage had statistical differences with recurrence. Although no significant differences were observed in multivariate logistic regression, it still suggests that the inflammatory levels may be related to the risk of recurrence. The importance analysis in the random forest model also indicated that, in addition to KAAS, blood pressure levels and inflammatory status (including postoperative complication occurrence and the proportion of inflammatory cells in preoperative blood routine) also have some importance in the prediction model for recurrence risk. Previous studies have suggested that hypertension may be involved in the development of keloids, and patients with hypertension often exhibit more severe keloid manifestations. There is a significant correlation between hypertension and the size and number of keloids (*p* < 0.0001) [18, 19]. Both postoperative complications (poor wound healing, surgical site infections) and the preoperative proportion of inflammatory cells represent the level of inflammation or immune status in patients. These factors are closely associated with the formation and development of keloids [20, 21, 22]. Therefore, these factors increase the risk of recurrence. KAAS has been previously shown to predict the recurrence of chest keloids [23]. This is a comprehensive scale for assessing the severity of keloids, including age, life habit, family history, course of the disease, speed of keloid growth, number of keloid lesions, hyperplastic halo, and Vancouver scar scale (Table 5).

**TABLE 5:** Keloid activity assessment scale (KAAS).

Items (score)	Indicators		score
Age (1)	≤ 40		1
	> 40		0
Life habit (1, in recent 2 years)	Meat	Much	0.25
		Less	0
	Vegetables	Much	0
		Less	0.25
	Water	Over 1.5 L a day	0
		Less 1.5 L a day	0.25
	Sleep	Sufficient(> 8 h/d)	0
		Insufficient(< 8 h/d)	0.25
Family history (2)	Yes		2
	No		0
The course of disease (1)	≤ 5 years		1
	5–10 years		0.5
	> 10 years		0
Speed of keloid growth (1)	Obvious growth (> 0.5 cm a year)		1
	No obvious growth		0
Number of keloid lesions (1)	≤ 5		0
	> 5		1
Hyperplastic halo (1) (Figure 1)	Yes		1
	No		0
Vancouver scar scale (1)	≥ 10		1
	< 10		0

*Note:* mild: < 4; moderate: 4–6.9; severe: 7–9.

A multivariable prediction model is defined as any combination of two or more predictor factors (variables, features) used to estimate the probability or risk that an individual has (diagnosis) or will develop (prognosis) a specific outcome [24, 25]. Correct and thorough reporting of predictive model studies is crucial for the proper implementation of the model in clinical practice.

The strength of ML lies in its ability to handle a large number of predictor variables. However, ML algorithms may ‘overfit’ predictions by capturing spurious correlations in the data. Any of these possibilities can lead to an overly optimistic estimation of model accuracy, and it must be addressed by testing the model on a truly independent validation dataset. Another critical issue is the quantity and quality of input data. ML algorithms heavily rely on data, and biases in data collection can significantly impact performance and generalizability [6]. ML algorithms are powerful tools in the healthcare domain, but sometimes they do not perform better than traditional models (such as linear regression, polynomial regression, ridge regression, etc.). Research on predictive models based on supervised ML suggests that, compared to traditional statistical techniques, predictive performance is more promising, and in some cases, even superior. However, some studies show contradictory results [26]. Many clinical studies have achieved satisfactory results using ML techniques, with AUC values ranging from 0.80 to 0.90, and sometimes even > 0.90. However, a high AUC is not necessarily a sign of high quality, as ML models can overfit. When traditional regression is applied and compared to ML algorithms, more complex ML models often provide only marginal accuracy gains. ML techniques undoubtedly provide powerful methods for handling predictive problems involving non‐linear or complex, high‐dimensional relationships. In contrast, many simple medical predictive problems are inherently linear, and feature selection is typically based on prior research, selecting these features because they are known strong predictors. One should acknowledge the limitations of ML algorithms and models in order to prevent their excessive utilization and misuse [27].

The limitations of this study include a small sample size, a single‐center design, a retrospective rather than prospective approach, and the absence of external validation. The limited sample size may affect the generalizability of our findings, as a larger and more diverse cohort could provide more robust insights. The single‐center design may introduce selection bias, limiting the applicability of our results to other populations or clinical settings. Additionally, the retrospective study design relies on previously collected data, which may lack the rigor and control of prospective studies. Finally, the absence of external validation restricts the ability to confirm the predictive performance of our models in independent datasets, which is a critical step for ensuring real‐world applicability. Addressing these limitations in future studies will be essential for strengthening the validity and clinical utility of our findings.

In the future, it is possible to establish a large‐scale database for keloids, incorporating additional features such as images and genetic sequencing results. There are currently existing research studies regarding ML image diagnosis for different scar categories [28]. The current Electronic Health Records (EHR) systems can be utilized to expand research centers and patient data [29]. Utilizing ML methods, this database could be used to predict the progression of keloids, enhancing our understanding of keloid treatment and prognosis. This advancement could potentially drive progress in clinical treatments for keloids.

## 5 Conclusions

In summary, three keloid recurrence prediction models were established on the basis of ML methods. It was found that factors such as KAAS, blood pressure levels, postoperative complications, and the proportion of inflammatory cells played significant roles in these models. Comparing the three models from various dimensions, in terms of predictive performance (AUC), the logistic regression model performed the best. The decision tree and random forest models could supplement factors not found in the logistic regression model.

## Author Contributions

Y.H. and Y.W. performed the research. M.S., H.L., and Y.W. designed the research study. Y.H. and X.K. contributed essential reagents or tools. Y.X. and K.S. analyzed the data. Y.H. wrote the paper.

## Ethics Statement

This study was approved by the ethics committee of the Institutional Review Board of Peking Union Medical College Hospital (Ethics Committee reference: S‐K1765).

## Consent

The authors have nothing to report.

## Conflicts of Interest

The authors declare no conflicts of interest.

## Data Availability

The data that support the findings of this study are available on request from the corresponding author. The data are not publicly available due to privacy or ethical restrictions.
